# Enhanced Antioxidant Activities of Metal Conjugates of Curcumin Derivatives

**DOI:** 10.1155/MBD.2001.183

**Published:** 2001

**Authors:** Sabari Dutta, Anupa Murugkar, Nitasha Gandhe, Subhash Padhye

**Affiliations:** 1 Department of Chemistry University of Pune Pune 411007 India

## Abstract

Antioxidant properties of three Curcumin derivatives in which the 1,3-diketone system is appended with nitrogen and sulfur donors and their copper conjugates are examined for the first time. Metal conjugation seems to offer distinct advantages in radical scavenging activities of curcumin compounds.


### ENHANCED ANTIOXIDANT ACTIVITIES OF METAL CONJUGATES OF CURCUMIN DERIVATIVES

Sabari Dutta, Anupa Murugkar, Nitasha Gandhe and Subhash Padhye*

Department of Chemistry, University of Pune, Pune-411007,India.
<sbpadhye@chem.unipune.ernet.in> Fax: +91 -20 –5651728/5653899

### ABSTRACT:

Antioxidant properties of three Curcumin derivatives in which the 1,3-diketone system is appended with nitrogen and sulfur donors and their copper conjugates are examined for the first time. Metal conjugation seems to offer distinct advantages in radical scavenging activities of curcumin compounds.

### INTRODUCTION:

Curcumin, 1,7-bis (4 hydroxy-3-methoxy phenyl)-1,6 heptadione-3,5-dione or diferuloyl methane, is a yellow pigment obtainable from the rhizomes of the plant *Curcuma longa*[1] , which is one of the major spices in the Indian curry powder. It exhibits a variety of pharmacological activities including anti-inflammatory, anticarcinogenic, antibacterial and antifungal activities[2-8] most of which are attributed to its antioxidant and radical scavenging properties[9-13]. The antioxidant activity of curcumin is influenced by the redox state of the biological environment[14], presence of metal ions and substituents on the side chain[12].

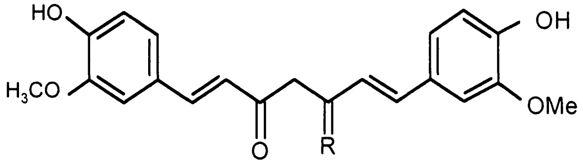

R = O, Curcumin (**L1**)
NOH, Curcuminoxime (**L2**)
$NNHCONH_2$, Curcuminsemicarbazone (**L3**)
$NNHCSNH_2$, Curcuminthiosemicarbazone (**L4**)

Earlier studies on curcumin have shown that the parent 1,3-diketone system and substituents on the phenolic side chain are important structural features that contribute to its antioxidant properties. Although Nair and Rao have examined the effect of phenolic substituents on the radical scavenging activity of various curcuminoids,[12] the influence of the modification of 1,3-diketone system has remained unexplored. The present paper thus examines the superoxide dismutating activity of the copper conjugates of three curcumin derivatives, which incorporate oxime, semi- and thiosemicarbazone functionalities in the curcumin nucleus.

### EXPERIMENTAL:

All chemicals used in the preparation of ligands and their metal complexes were AR grade or its equivalent and were used without further purification. Solvents used in the syntheses were purified prior to their use according to literature methods[15]. Curcumin was the product of Aldrich while hydroxylamine hydrochloride, semicarbazide hydrochloride, $CuCl_2.H_2O$ were obtained from SD Fine Chemicals, Bombay, Thiosemicarbazide (BDH) was converted into its hydrochloride by known method[16].

*1. Preparation of Curcuminoid Ligands L2 - L4:*

Curcumin derivatives, viz. **L2-L4** were prepared according to the standard organic procedures described by Vogel[17].

*2. Preparation of Cu (II) complexes of L1 – L4:*

Copper (II) complexes of curcumin derivatives were synthesized by reacting methanolic solutions of the ligands with an aqueous solution of $CuCl_2.H_2O$ under reflux for 3 hr in the stoichiometric ratio of 1:2 (metal:ligand) for **L1** and **L2**, while 1:1 (metal:ligand) for **L3** and **L 4** complexes. The precipitated complexes were filtered, washed with cold water and ethanol and dried in vacuum.

*3. Physico-chemical measurements*

The magnetic susceptibilities of the synthesized complexes were measured at room temperature on a Faraday type magnetic balance having a field strength of 7000 Gauss. The molecular susceptibilities were corrected for diamagnetism of the component atoms using Pascal's constants[18]. The apparatus was calibrated using mercury tetrathiocyanatocobaltate as a reference standard[19]. Infrared spectra of the ligands and their metal complexes were recorded as nujol mulls on Perkin-Elmer FT-IR 283-B instrument while UV-VIS spectra were measured on Genesys-2 machine using quartz cells. The electrochemical measurements were made in DMF solvent using tetraethylammonium perchlorate (TEAP) as the supporting electrolyte with the help of BAS cyclic voltammetric automatic system CV-27 under dry nitrogen atmosphere. The three electrode system employed consisted of platinum working electrode, platinum wire as auxiliary electrode and SCE as the reference electrode. The ESR spectra were recorded in DMSO at 77K on Varian E-112 X-band spectrophotmeter at RSIC, IIT Powai, Mumbai, (India).

*4. Superoxide Dismutase (SOD) assay:*

The SOD activity of the curcuminoid ligands and their copper conjugates was determined spectrophotometrically by the NBT assay employing Xanthine/Xanthine oxidase as the source of super - oxide radical[12]. One unit of SOD activity was defined as the concentration of the test substance required for 50% inhibition of NBT reduction by the superoxide ($IC_{50}$ value) anion[20].

**RESULTS:**

The interaction of $CuCl_2$ with **L1** and **L2** yields compounds having composition $CuL_2$ while in case of **L3** and **L4** the stoichiometry of the resulting complexes is found to be [Cu(L)Cl] respectively. All complexes are found to be diamagnetic at 298 K indicating essentially a planar geometry for these compounds[21] as shown in **Figure 1a and 1b**.

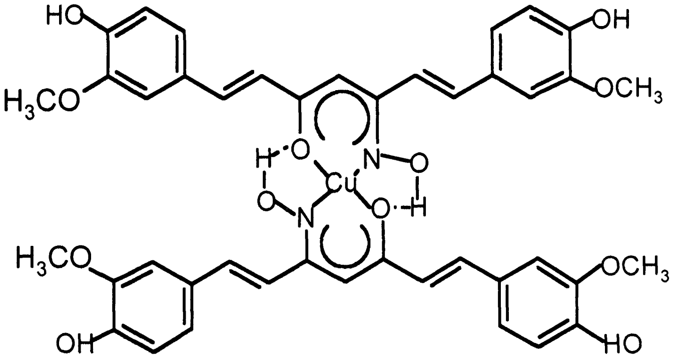

**Figure 1a**: Proposed structure of the complex $[Cu(L1)_2]$

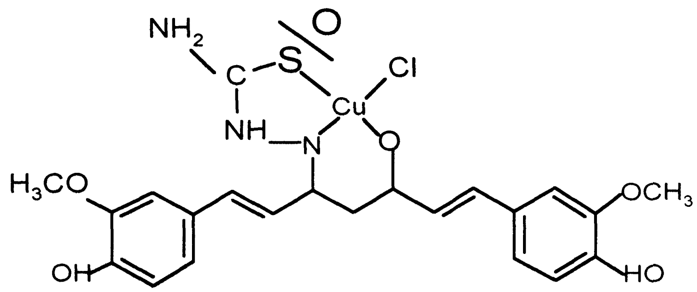

**Figure 1b**: Proposed structure of the complex [Cu(L2)Cl] & [Cu(L3)Cl]

The IR spectra of **L1** is characterized by the presence of a single strong band at 1745 $cm^{-1}$ due to the central carbonyl absorptions functionalities which are equivalent[22]. The generation of the oxime functionality at one of the carbonyls can be ascertained from the presence of imine band at 1587 $cm^{-1}$ and a strong carbonyl absorption at 1628$cm^{-1}$ respectively[23]. On metal complexation both the bands are shifted to lower frequency indicating their involvement in copper complexation. The introduction of semi- and thiosemicarbazone functionalities in the curcumin nucleus is best diagnosed from the symmetric and asymmetric bands around 3200-3400 $cm^{-1}$. For the semicarbazone species the amide carbonyl absorption can be observed at 1627 $cm^{-1}$, which on copper complexation completely disappears and is replaced by a band at 1207 $cm^{-1}$ indicative of a phenolic ν(C-O) frequency. In case of the ligand **L4** the to thiocarbonyl stretching frequency is seen at 1276 $cm^{-1}$ which undergoes a considerable shift to lower frequency side ($\Delta$ = 66$cm^{-1}$) on metal complexation indicating its participation in metal coordination. In compounds **L3** and **L4** the curcumin carbonyls are observed around 1580-1590 $cm^{-1}$ while the imine absorption is seen at 1510-

1520 $cm^{-1}$ respectively. Both of these absorptions are found affected by copper conjugation and are found shifted to lower wavenumbers.

| Molecular Formula | % C (Calc.) Obsd. | % H (Calc.) Obsd. | % Cu (Calc.) Obsd. | $E_{1/2}$ (V) $Cu^{+/2+}$ | $g_{\|\|}$ | $g_{\perp}$ | F = $g_{\|\|}/A_{\|\|}$ (cm) |
|---|---|---|---|---|---|---|---|
| [Cu(**L1**)$_2$] $C_{42}H_{38}O_{12}Cu$ | (62.97) 63.81 | (4.75) 5.14 | (8.14) 7.95 | + 0.45 | 2.144 | 2.078 | 147.72 |
| [Cu(**L2**)$_2$] $C_{42}H_{40}O_{12}Ncu$ | (61.84) 60.77 | (4.13) 4.83 | (7.17) 7.66 | + 0.42 | 2.425 | 2.053 | 152.96 |
| [Cu(**L3**)Cl] $C_{22}H_{22}O_6N_3ClCu$ | (50.36) 50.52 | (4.21) 4.19 | (12.85) 12.12 | + 0.37 | 2.380 | 2.066 | 157.19 |
| [Cu(**L4**)Cl] $C_{22}H_{22}O_5N_3SClCu$ | (49.02) 48.86 | (4.29) 4.07 | (12.19) 11.76 | + 0.41 | 2.460 | 2.080 | 154.90 |

**Table 1.** Analytical data on copper(II) complexes of L1-L4 including cyclic Voltammetric and ESR data

The electronic spectra of curcumin derivatives show prominent absorptions at ~ 266 nm and 428 nm due to the $\pi$ - $\pi^*$ transitions respectively[24] which undergo minor shifts on metal complexation indicating that no major structural alteration occurs during complexation. Interestingly metal complexes of **L2** – **L4** exhibit intense and predominant charge transfer bands around 434 nm resulting in masking of the weaker d-d transitions[24].

The ESR spectra of all copper complexes are typical of axial symmetry with $g_{||}>g_{\perp}>2.0$ indicating the presence of electron in $d_{x^2-y^2}$ ground state.

The cyclic voltammetric profiles of all ligands and of their copper complexes were studied in DMF. Curcumin shows one reversible peak centered at –0.84 V ascribed due to the reduction of its carbonyl functions[25]. On derivatizing with the imine function this peak is shifted to –0.68V and its reversibility is lost (**Figure 2**). A reversible $Cu^{2+}/Cu^{+}$ redox couple is observed in the copper conjugates of **L1 to L4** between + 0.37 to + 0.45 V.

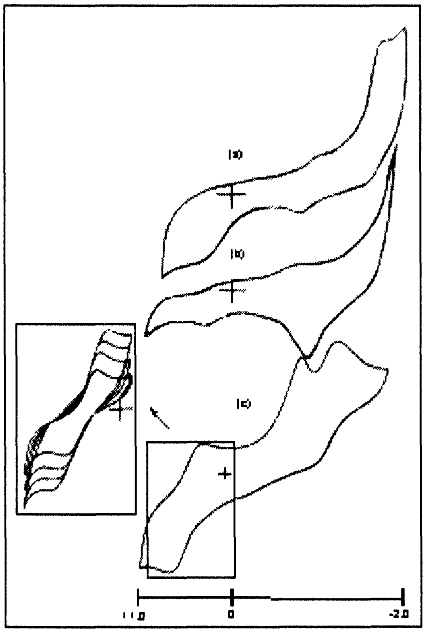

**Figure 2:** Cyclic voltammograms of (a) 0.1 M **L1** (b) 0.1 M **L3** (c) 0.1 M [Cu(**L3**)Cl] in 0.1 M TEAP in DMSO. Inset shows the scan rate dependence for the peak centered at + 0.37 V of [Cu(**L3**)Cl].

**SOD Activity:**

The results of the SOD assay on curcumin derivatives and their metal complexes **(Figure. 3)** reveal that the parent ligand and its copper conjugate exhibit antioxidant activity at higher concentrations, while all imine derivatives (**L2** to **L4**) and their copper complexes show potent antioxidant activities at much lower concentrations indicating that copper conjugation enhances the antioxidant property of the parent Schiff base ligands remarkably, the most potent compound being the copper conjugate, **L3,** with $IC_{50}$ value of 0.28 μM.

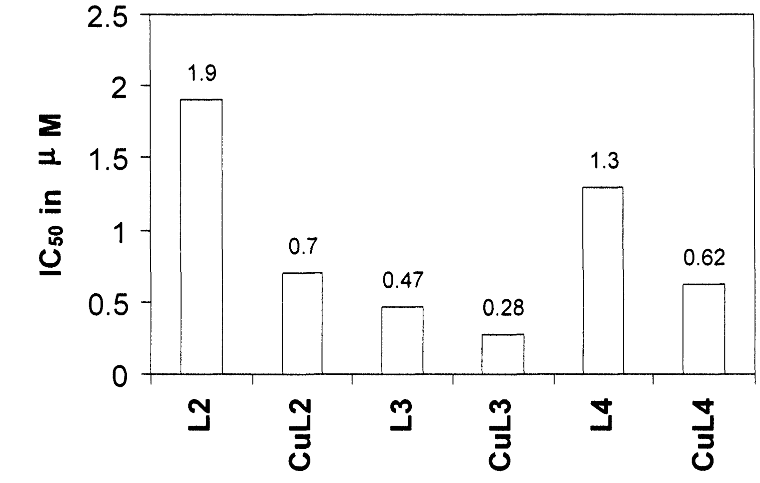

**Figure 3**: The $IC_{50}$ values in μM of the curcumin derivatives and their metal complexes

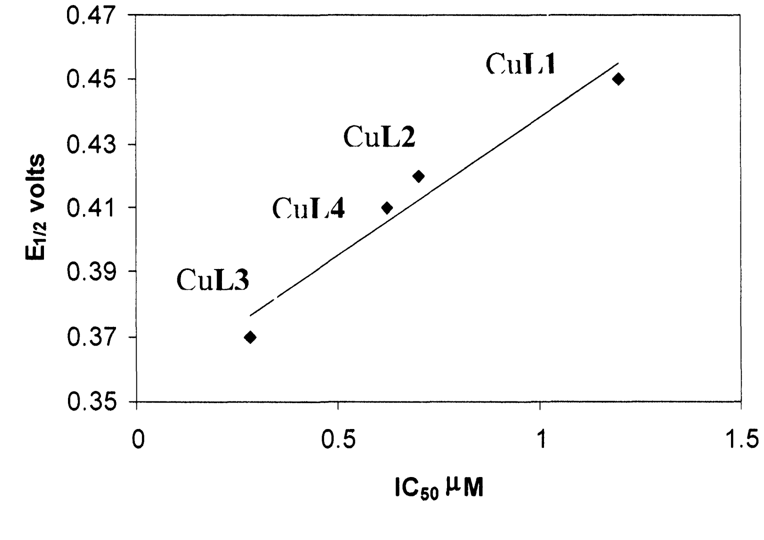

**Figure 4** : The $IC_{50}$ values of the copper curcumin derivatives versus their redox potentials.

This value compares well with other synthetic SOD mimics reported in literature recently[26]. It is interesting to note that tridentate derivatives such as the Schiff bases of **L3** and **L4** turn out to be the most potent SOD mimics. When the redox potentials ($E_{1/2}$) of the copper conjugates are plotted against the $IC_{50}$ values of their SOD activity, a linear relationship becomes apparent (**Figure 4**) suggesting that $E_{1/2}$ values in the present case correlate well with the percent distortion from the planarity[27] (**Figure 5**) which is another parameter suggested by Cao et.al. for such structure-activity correlations[28].

### CONCLUSIONS:

The present work has thus shown that copper conjugates of the Schiff base derivatives of curcumin yield highly potent SOD mimics. The observed correlation between the SOD activity and the redox potential of the $Cu^{+}/Cu^{2+}$ emphasizes the roles played by the electronic[29] as well as stereochemical factors in the biological activities[30] of these curcuminoids.

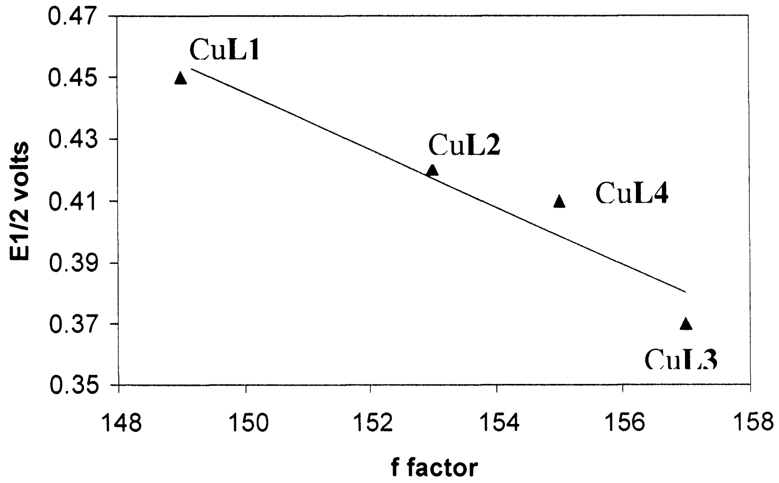

**Figure 5 :** The f factor of the copper curcumin derivatives versus their redox potentials.

### REFERENCES:

1. H.Ahsan, S.M. Hadi, , *Cancer Lett.* **124**, 23 (1998).
2. S.P.Verma, E.Salamone, B. Goldin, *Biochem.and Biophys.Res.Comm.* **233**,692 (1997).
3. E.Kunchundy, M.N.A. Rao, *Int.J.Pharmacol.***57**, 173 (1989).
4. B. Vijaylaxmi, *Mutat. Res* **79**, 125 (1980).
5. T.A.Dahl, W.M.McGowan, M.A.Shand, V.S. Srinivisan, *Arch.Microbiol,* **151**,183 (1989).
6. M .Nagabhushan, S.V. Bhide, *J.Am.Coll.Nut,.* **11**,192 (1992).
7. H.P.T. Ammon, W.A. Wahl, *Planta.Med,* **57**, 1 (1991).
8. O.P. Sharma, *Pharmacol,* **25**,1811 (1976).
9. E. Kunchundy, M.N.A. Rao, *Int.J. Pharmacol,***58**, 237 (1990).
10. S.K.Kim, D. Han. K.D. Moon, and J.S. Rhee, *Biosci.Biotech.Biocehm,***59**, 822 (1995)
11. S.Toda, T. Mujase, H Archi, H.Tanizawa, H. Takino, *Chem.Pharna.Bull,* **33**, 1725 (1985).
12. S.Nair, M.N.A. Rao, *Drug Research,* **46**, 169 (1996).
13. K.I. Priyadarsini, *Free.Rad.Bio. and Med,* **23**, 838 (1997).
14. S.C.Sahu, M.C. Washington, *Cancer Lett.***63**, 237 (1992).
15. D.D.Perrin, W.L.. Armargo and D.R. Perrin, *Purification of Laboratory Chemicals.* Pergamon Press, London: 1966.
16. P.B. Sonawane Ph.D. Thesis, University of Pune (India), 1992.
17. B.S.Furniss, A.J. Hanneford, W.G. Smith, and A. R.Tatchell,: *Vogel's Text book of Practical Organic Chemistry,* 5th Edition, Longman Publishers Pvt. Ltd. : 1989.
18. B.N. Figgis, and J. Lewis, *Modern Coordination chemistry, Principles and Methods'*,Ed. Lewis J. and Wilkinsone, R.J. Interscience, New York (1968), pp 403.
19. R.S. Nyholm , and Figgis, *J.Chem.Soc,* 4190 (1958).
20. R.L.Arudi, A.O. Allen, and B.H.J.Bielski, *FEBS Lett.* **2**,135 (1981).
21. R.L. Dutta & A.Syamal, '*Elements of Magnetochemistry,*' 2nd Ed, Affiliated East West Press Pvt. Ltd., (1993)
22. K.K. Sharma, S.Chandra, *Inorg.Chim.Acta,* 135,47 (1987).
23. J.Charalmbous,; M.J. Frazer, and F.B.Taylor, *J.Chem.Soc (A)*: 2787 (1969).
24. (a) Y.Nishida, S. Kida, *Coord.Chem.Rev,* **27**, 275 (1979).(b) A.B.P Lever, *Inorganic Electronic Spectroscopy,* 2nd: Elsevier, Amsterdam, 1984.
25. A.S.Kumbhar, S.B. Padhye, D.X.West, and A.E Liberta, *Trans.Met.Chem,* **16**,176 (1991).
26. G.Tabbi, W.L.Driessen, J. Reedijk, R.P. Bonomo, N, Veldman, L. Spek, *Inorg. Chem.,* **36**,1168 (1997).

27. A.Diaz, R. Cao, A. Fragoso, I. Sanchez, *Inorg.Chem.Comm.***2** , 358, (1999).
28. Z. Durackova, M.A. Mendiola, M.T. Sevilla, and A Valent, *Bioelec.Bioener*, **48**,109(1999).
29. (a) R.P.Bonomo, E Conte, R .Marchelli, A.M .Santoro, G.J.Tabbe, *J.Inorg.Biochem*,**53**,127 (1994).
30. (a)R.P Bonomo, A .Deflora, E. Rizzarelli, A.M. Santiono, G.Tabbi, M.J. Tonetti, *J.Inorg.Biochem*, **59**,773(1995). (b) R.P Bonomo, R.Marchelli, G. Tabbi, *J.Inorg.Biochem*, **60**, 205 (1995).

**Received: September 18, 2001 – Accepted: October 18, 2001 – Accepted in publishable format: October 19, 2001**

